# Exercise in adults admitted to hospital with diabetes-related foot ulcers: a pilot study of feasibility and safety

**DOI:** 10.1186/s13047-023-00616-0

**Published:** 2023-03-28

**Authors:** Emily Aitken, Jonathan Hiew, Emma J Hamilton, Laurens Manning, Jens Carsten Ritter, Edward Raby, Paul M Gittings

**Affiliations:** 1 Physiotherapy Department, Fiona Stanley and Fremantle Hospital Group, Murdoch, Western Australia; 2 Podiatry Department, Fiona Stanley and Fremantle Hospital Group, Murdoch, Western Australia; 3 Multidisciplinary Diabetes Foot Unit, Fiona Stanley Hospital, Murdoch, Western Australia; 4 Endocrinology Department, Fiona Stanley and Fremantle Hospital Group, Murdoch, Western Australia; 5 School of Medicine, University of Western Australia, Perth, Western Australia; 6 Infectious Diseases and Microbiology Department, Fiona Stanley and Fremantle Hospital Group, Murdoch, Western Australia; 7 Vascular Surgery Department, Fiona Stanley and Fremantle Hospital Group, Murdoch, Western Australia; 8 School of Medicine, Curtin University, Perth, Australia

**Keywords:** Exercise, Exercise therapy, Diabetic foot, Foot ulcer, Diabetes mellitus

## Abstract

**Background:**

Diabetes-related foot ulcers result in significant mortality, morbidity and economic costs. Pressure offloading is important for ulcer healing, but patients with diabetes-related foot ulcers are presented with a dilemma, because whilst they are often advised to minimise standing and walking, there are also clear guidelines which encourage regular, sustained exercise for patients with diabetes. To overcome these apparently conflicting recommendations, we explored the feasibility, acceptability and safety of a tailored exercise program for adults admitted to hospital with diabetes-related foot ulcers.

**Methods:**

Patients with diabetes-related foot ulcers were recruited from an inpatient hospital setting. Baseline demographics and ulcer characteristics were collected, and participants undertook a supervised exercise training session comprising aerobic and resistance exercises followed by prescription of a home exercise programme. Exercises were tailored to ulcer location, which complied with podiatric recommendations for pressure offloading. Feasibility and safety were assessed via recruitment rate, retention rate, adherence to inpatient and outpatient follow up, adherence to home exercise completion, and recording of adverse events.

**Results:**

Twenty participants were recruited to the study. The retention rate (95%), adherence to inpatient and outpatient follow up (75%) and adherence to home exercise (50.0%) were all acceptable. No adverse events occurred.

**Conclusions:**

Targeted exercise appears safe to be undertaken by patients with diabetes-related foot ulcers during and after an acute hospital admission. Recruitment in this cohort may prove challenging, but adherence, retention and satisfaction with participation in exercise were high.

**Trial registration:**

The trial is registered in the Australian New Zealand Clinical Trials Registry (ACTRN12622001370796).

**Supplementary Information:**

The online version contains supplementary material available at 10.1186/s13047-023-00616-0.

## Introduction

Pressure offloading is a critical component of a multidisciplinary management plan to achieve diabetes related foot ulcer (DFU) healing [1]. The mechanism for this is through redistribution or elimination of the forces associated with ambulation and weight bearing which can be achieved by the usage of specific offloading devices. In addition to offloading devices, patients are often advised to minimise physical activity and exercise [2, 3]. However, physical activity and exercise are important aspects of diabetes management via several physiological benefits not limited to improved glycaemic control and stability, increased lean muscle mass, improved muscle strength, reduced mortality and improved quality of life [4–9].

As such, people living with diabetes-related foot ulcers (DFU) are often faced with a dilemma on what role physical activity and exercise has in the overall management plan for diabetes in the context of a DFU. In addition to the known benefits of physical activity and exercise, lower levels of physical activity in patients with diabetes has been shown to be associated with foot ulceration [10, 11] supporting the importance of participation in physical activity and exercise in this patient group.

Exercise programmes undertaken by people with diabetes-related peripheral neuropathy, with and without existing foot ulcers, do not increase the rate of ulceration [2, 11–14]. Instead, the physiological benefits of exercise may include improvements in neuropathic symptoms, nerve fibre branching and glycated haemoglobin (HbA1c) levels [2, 12–14]. One small study suggested that non-weight bearing exercise is safe and feasible for people with DFU in an ambulatory care setting [15]. It did not however explore the role of targeted exercise beyond non-weight bearing exercises. Recent systematic reviews have concluded that that there is insufficient high quality evidence for a conclusive outcome about the impact of exercise on patients with DFU, or DFU healing [16, 17]. Recommendations are for further research in this area.

This knowledge gap has been identified by The International Working Group on the Diabetic Foot (IWGDF) as an important area for future research and was amongst the top ten research priorities for Australian stakeholders which included consumers and health professionals [18]. It is known that every two hours, an Australian loses a limb to a diabetes-related foot ulcer (DFU) [19] with significant morbidity and economic costs. As such, investigation into interventions that may assist to reduce complications of DFU is an important endeavour. To assist in clarifying apparently conflicting physical activity and exercise recommendations in this vulnerable group, we aimed to determine whether undertaking exercise in people admitted to hospital with an acute DFU is feasible, acceptable and safe. Secondary aims included physical activity levels, perceived benefits and barriers to physical activity, muscle strength, exercise load and satisfaction with the exercise programme.

## Methods

### Study Design

This non-randomised pilot study aimed to determine safety and feasibility of targeted exercise commencing during an acute inpatient setting. Ethics approval was granted by the South Metropolitan Health Service Human Research Ethics Committee (RGS 4173). Study data were collected and managed using Research Electronic Data Capture (REDCap) [20, 21].

### Participants

Patients admitted to hospital under the care of the Multidisciplinary DFU team between October 2020 to April 2021 were screened for eligibility. Participants were eligible if they were over the age of 18, had a diagnosis of diabetes mellitus and requiring admission to hospital for DFU of any type or severity. People were excluded from participation if they were unable to consent due to language or cognitive impairment, had an acute myocardial infarction, unstable angina, severe heart failure (New York Heart Association Functional Classification IV), cardiac arrhythmias causing haemodynamic compromise, musculoskeletal or neurological conditions precluding exercise or it was otherwise determined not to be in their best interest to participate. Written consent was obtained for all participants. The resources available for this study limited patient screening and recruitment to one day per week.

### Study procedures

Demographic information was collected, surveys and physical assessments (described in Outcomes section below) were completed. Participants completed an individually prescribed inpatient exercise session under supervision. Following this, they were provided with an individualised daily home exercise programme to complete for two weeks post discharge (see Intervention section below). Podiatric wound offloading recommendations were observed at all times during this study.

After completion of the inpatient phase of the study, participants were reviewed at their routine outpatient follow-up clinic, scheduled for two weeks after discharge. Adherence to their home exercise programme was assessed by review of a home exercise diary. Final surveys and physical outcome measurement were also completed. Participants were also offered an outpatient exercise session at this review. Participants who could not attend in-person after discharge were followed up via telephone and surveys were emailed to participants for completion using REDCap. Upon exit from the study, participants were provided a short survey asking about acceptability and satisfaction of the exercise intervention. Adverse events, participant comments and any reasons for withdrawal were collected throughout the study.

### Intervention

Exercise was commenced during hospital admission. Where a participant required surgery for their DFU, clearance from the treating team was obtained for the patient to commence exercise. The treating team made weight bearing orders in relation to the DFU location for each participant to follow. Participants who were able to weight bear but in offloading footwear were prescribed this by the Podiatrist. These included removable ankle high and knee high devices. Otherwise, participants who were ordered to be non-weight bearing were able to participate in exercise whilst adhering to this restriction. Blood glucose levels (BGL) were assessed prior to and during exercise sessions. Exercise was not commenced, or was stopped, where BGL < 5 mmol/L.

Supervised exercise sessions were comprised of both aerobic and strength training components. These exercises were individualised for each participant to maintain weight bearing orders given by the treating team. For example, participants who were ordered to be heel weight bearing undertook exercises weight bearing through the heel only. Participants ordered to be toe weight bearing completed exercises weight bearing through the toe only. Where a patient was strictly non-weight bearing on one leg, exercises were performed weight bearing on the opposite leg and non-weight bearing on the affected leg. Exercise selection was completed with respect to the participant’s ability to achieve the full range of movement of the selected exercises. This was assessed at the time of exercise by the supervising Physiotherapist.

The mode of aerobic exercise was selected using a combination of patient preference and adherence to wound offloading requirements. Participants completed five to 20 min of upper limb ergometry (Monark Exercise AB, Dalarna, Sweden), single leg or two-legged cycling on an upright exercise bike (Monark Exercise AB, Dalarna, Sweden), or recumbent exercise bike (SportsArt, Washington, United States of America). The amount of time for the aerobic component was based on exercise tolerance. Participants were instructed to exercise at a moderate intensity using Borg’s Rating of Perceived Exertion Category-Ratio scale (CR-10) [22].

Strength training exercises were completed as a circuit using body weight, resistance bands (Performance Health ANZ, Sydney, Australia), free weights and ankle weights, or pin-loaded cable weight machines (Cybex International, Illinois, USA). Exercises were selected based on weight bearing orders as described above. Participants were instructed to work to a moderate intensity on the CR-10 scale. Each participant completed a total of 2–3 sets of 8–15 repetitions of each exercise. Exercises targeted key muscles groups in both lower and upper limbs in standing, sitting or unilateral positions. Examples of some of the common exercises utilised were; bicep curl, shoulder press, deltoid fly, seated or bent over row, leg press, sit to stands, squats, bridging, heel raise, side lying or standing hip abduction and seated knee extensions. Participants were asked to rate the intensity of the full exercise session using the CR-10 scale at 10 min after their session.

A home exercise programme was prescribed and consisted of a selection of pre-determined exercises similar to those utilised in the inpatient session (Additional file 1). These accounted for individual ability and wound offloading requirements. These exercises were aimed at strength training and utilised hand weights (or water bottles / other common household items) or resistance bands (Performance Health ANZ, Sydney, Australia) which were provided. Home exercises were demonstrated by a Physiotherapist who ensured these were able to be completed safely prior to discharge. A home exercise diary was provided to participants and checked at the two week follow up. Where a patient was unable to attend in person, this was reported subjectively on the phone to the researcher.

### Outcomes

#### Primary outcome

The primary outcome of this study was the feasibility and safety of undertaking exercise in the study population (Table 1) and was assessed at the end of the study. These criteria and acceptability limits were agreed upon by the study team during development of this project.

**Table 1:** Feasibility and safety outcome measurement and acceptability levels

Outcome	Description	Acceptability
Recruitment	Number of participants enrolled in the project as a proportion of all eligible participants approached for consent	50% patient recruitment
Retention	Number of participants who remained enrolled in the project	75% retention
Adherence to study	Number of participants who completed both inpatient and outpatient phases of the project	75% adherence
Adherence to home exercise	Completion of home exercise programme	Any amount of completion
Adverse Events	Safety will be assessed by examination of the reported adverse events related to exercise participation	N/A

Adverse events in this study were defined as BGL < 5.0mmol/L during exercise, any event related to the exercise session which required a referral for an unplanned medical review, unplanned repeat debridement of index ulcer during the enrolment period or unplanned amputation involving the index ulcer during the enrolment period.

#### Secondary outcomes

##### Current levels of physical activity

Current participation in physical activity and exercise was assessed using the International Physical Activity Questionnaire (IPAQ) - short. This is a seven-item questionnaire which can be self-administered, or telephone administered. It is a validated tool to obtain data on health-related physical activity [23]. The questionnaire quantifies how much vigorous, moderate or walking based activity on an average week and converts this to a weighted estimate of total physical activity. This estimate is then used to classify participant’s physical activity level as low, moderate or high [23]. This was assessed at initial review and two week follow up.

##### Benefits and barriers to physical activity

Perceived benefits of and barriers to physical activity and exercise was assessed using the Exercise Benefits/Barriers Scale (EBBS) [24]. This is a 43 item four-choice Likert scale in which the respondent rates their agreement with perceived barriers or benefits of exercise [24, 25]. The EBBS can be scored as a Benefits scale and a Barriers scale, where a higher score indicates the responder has greater perception of benefits or barriers to exercise respectively. In addition to the EBBS, we asked participants whether they believed that having a foot ulcer was a barrier to them participating in exercise. This was as a “yes/no” question format. This was assessed at initial review and two week follow up.

##### Muscle strength

Grip strength was assessed in kilograms using a Jamar handheld dynamometer (Surgical Synergies, SI Instruments, SA, Australia). Grip strength has been demonstrated to be useful as a predictor for muscle mass and physical functioning [26]. This was assessed at initial review and two week follow up.

##### Exercise Intensity and load

The intensity of exercise was rated using the CR-10 scale. This is a 0–10 scale used to grade exercise intensity [27]. This scale allows individuals to rate their exertion and monitor the intensity of exercise. The overall intensity of the exercise session was assessed by asking the patients for a session RPE (sRPE) score to quantify the intensity of the exercise session [28]. The sRPE is a multiplication of the intensity score for the session as a whole by the duration of the session in minutes. This was assessed 10 min after at the end of an exercise session.

##### Satisfaction

An exit survey was given to participants at the end of enrolment in this study asking about the participant’s satisfaction with the intervention. It was based on previous research and investigated participant’s perceptions of benefit and safety [29]. A five-point Likert scale was used asking participants to rate their level agreement with the following statements, from strongly disagree [1] to strongly agree [5]:

1. I found the exercise sessions in hospital useful
2. I felt safe completing exercise in the hospital
3. I felt that the supervision with exercise in hospital was adequate
4. I was able to do the home exercise programme easily in my home
5. I found the home exercise programme useful
6. I would recommend participation in an exercise programme like this

### Sample size

The enrolment target was 30 participants.

### Statistical methods

Descriptive statistics were used to describe the demographic data, primary and secondary outcomes. Primary outcomes were compared to pre-determined criteria displayed in Table 1. Paired data from 15 participants who completed both study phases for the EBBS and IPAQ were analysed. The EBBS data were compared using a Wilcoxon signed-ranked analysis whilst data from the IPAQ was compared using a chi-square analysis. We have chosen to display the Likert data in chart form to highlight the frequency of answers in these fields.

## Results

**Fig. 1:**
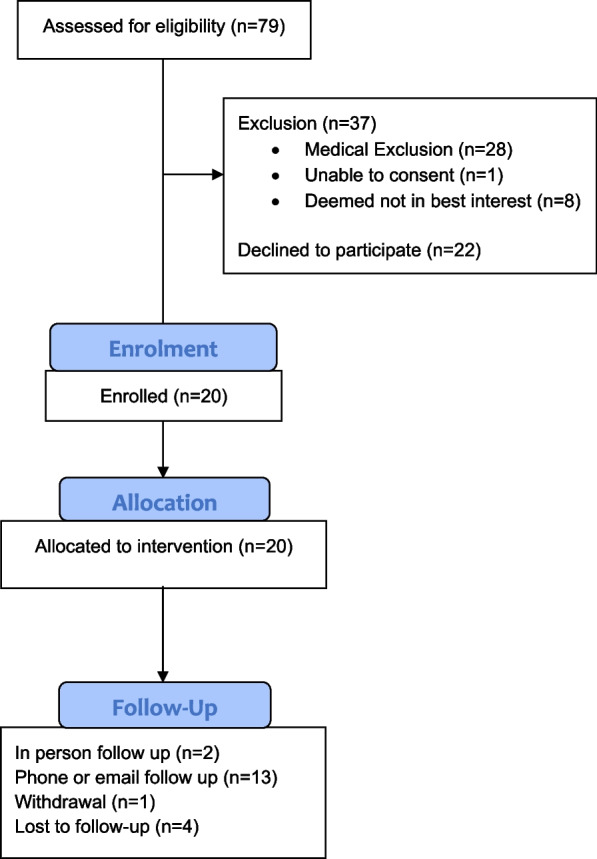
Flow of participants through the study

Forty-two patients were identified as suitable candidates and 20 patients provided consent and were enrolled into the study. Fifteen participants continued through the study and provided end-study data. The flow of participants through this study is presented (Fig. 1). The most common reasons for declining to participate are shown (Table 2).

**Table 2:** Reasons for declining to participate

Reason	n
Participant feels they do enough exercise already	5
Declined to give a reason	5
Participant not feeling well enough to exercise	4
Participant is for imminent discharge from hospital	4
Participant feels it is too much of a time commitment	2
Participant does not like exercise	1
Participant worried about the impact of exercise on DFU	1

Demographic data of enrolled participants are shown (Table 3). The exercise intervention was conducted before planned surgical intervention of the foot ulcer in 12 (60%) of the participants and after planned surgery for 5 (25%) of the participants. Wound offloading footwear was prescribed for 11 (55%) of the participants. Six participants (30%) were ordered to be non-weight bearing on the affected limb and subsequently undertook non-weight bearing exercises for that limb.

**Table 3:** Demographic and foot ulcer characteristics of the study population

	n (%)
Age [mean (SD)]	61.3 (12.3) years
BMI^a^ [mean (SD)]	29.9 (5.2) kg/cm^2^
Duration of diabetes [mean (SD)]	21.3 (12.5) years
HbA1c^b^ [mean (SD)]	8.82 (2.23)
Female	7 (35)
Diabetes	
- Type 1	2 (10)
- Type 2	18 (90)
Smoking History	
- Never smoked	8 (40)
- Previous smoker	8 (40)
- Current smoker	4 (20)
Chronic Kidney Disease	7 (35)
Cardiovascular Disease	7(35)
Musculoskeletal Disease	16 (80)
Previous foot ulcer	14 (70)
Ulcer Location:	
- Forefoot	15 (75)
- Midfoot	3 (15)
- Hindfoot	2 (10)
WIfI^c^ wound grade [30]	
- 1	8 (40)
- 2	7 (35)
- 3	5 (25)

Data is presented as [number (%)] unless otherwise informed described.

Key

^a^Body mass index

^b^Hemaglobin A1c

^c^Wound, ischemia and foot infection

### Primary outcomes

Feasibility and safety data is presented in Table 4.

**Table 4:** Primary outcome feasibility and safety outcome data

Outcome	n	%
Recruitment	20 of 42	47.6
Retention	19 of 20	95.0
Adherence to study	15 of 20	75.0
Adherence to home exercise	10 of 20	50.0
Adverse Events	0	0.00

### Secondary outcomes

**Fig. 2:**
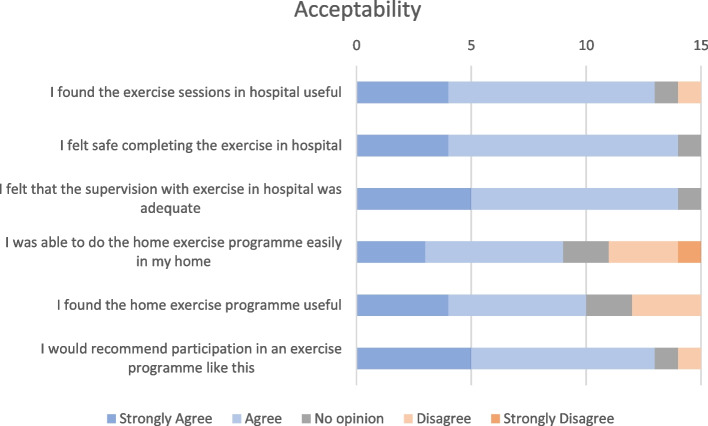
Post treatment acceptability of participating in an exercise intervention (*n*=15). Participant responses to questions regarding acceptability of participating in an exercise intervention, as provided in the exit survey. Values represent frequency of responses for each question

The secondary outcomes for the initial and final assessment time points are presented in Table 5, for participants that completed both phases of the study. The data for acceptability and satisfaction of the exercise programme are presented in Fig. 2.

**Table 5:** Secondary outcomes

Outcome	Initial Assessment (*n* = 20)	Final Assessment (*n* = 15)	*p*-value
IPAQ [n (%)]			
- High	2 (13)	2 (13)	0.999
- Moderate	3 (20)	3 (20)	
- Low	10 (67)	10 (67)	
EBBS [mean (SD)]			
- Benefits Scale	89.1 (11.8)	82.8 (10.5)	0.003
- Barriers Scale	30.9 (5.5)	30.3 (5.7)	0.705
Perception that a foot ulcer prevents exercise participation [n (%)]	11 (73)	7 (47)	
Grip strength [mean (SD)]	Left: 28.3 (11.0) Right: 31.8 (13.6)		
Exercise Intensity RPE^a^ [mean (SD)]		3.5 (1.07)	
Exercise Session Training Load sRPE^b^ [mean (SD)]		151.5 (67.7)	

Key:

^a^ Rating of perceived exertion

^b^ Session rating of perceived exertion

## Discussion

The undertaking of an exercise intervention in a patient group hospitalised with a DFU was a novel feature of this pilot study. We chose to include patients during their acute hospital admission for a DFU as an opportunity to provide enhanced multidisciplinary care during the early, active phase of treatment. The results of this study suggest that the inclusion of an exercise programme is feasible and safe for people hospitalised with a DFU.

No adverse events occurred during exercise sessions. Providing supervision during exercise sessions was believed to be key in achieving this safety outcome. Supervision allowed for monitoring of vital clinical parameters (such as BGLs, heart rate and blood pressure) as well as subjective parameters (such as level of exercise intensity), thereby minimising potential risks associated with over-exertion in this population. Furthermore, supervision enabled the authors to provide exercise specifically tailored to individual wound offloading needs. Previous research has trialled non-weight bearing exercises to achieve offloading requirements [15]. This study also utilised a range of common exercises with altered foot positions to comply with ulcer off-loading requirements, without restricting exercise options to only non-weight bearing exercises in participants who were able to weight bear to some degree. Whilst we were unable to assess plantar pressures in this study, assessment of this would confirm effectiveness of this approach.

Weight bearing is an important consideration in this patient group. Whilst it is a recognised mode of protection for wound healing, the off-loading of a limb comes with its own health risks. A recent study has demonstrated bilateral reductions in bone mineral density (BMD) of 1.4–2.8% at the femoral neck and total BMD at the hip 12 weeks after hospital admission for DFU [31]. The authors of this study concluded that this finding was likely to be related to disuse due to prolonged offloading periods, and elevated levels of serum inflammatory markers. Exercise training is an intervention that has been shown to be effective in the maintenance or improvement of BMD in a number of clinical and healthy groups [32]. Exercise training has also been demonstrated to be effective in the reduction of systemic inflammation for people with diabetes [33]. The effect of commencing exercise in the acute phase of an admission due to DFU on BMD loss could assist in ameliorating these impacts of DFU treatment. Whist the current study has provided evidence that exercise is feasible, the effectiveness of this relatively low cost and simple intervention is not known and should be considered for further investigation as it may have important clinical implications, including avoiding secondary complications of a DFU.

The potential for exercise and its role in reducing the risk of complications associated with DFU has been identified as a research priority by health providers and consumers [18]. There are however, a number of known barriers for participation in exercise for this patient group that should be considered [12]. The current study demonstrated that the presence of a DFU was viewed as a barrier to exercise participation by the majority of participants at the commencement of the study. The demonstration of a lower percentage of participants viewing their DFU as a barrier to exercise at the end of the study period suggests that demonstrating simple modification to a variety of common exercises could be an effective way to reduce the belief of a DFU as a barrier to exercise. In clinical practice, reducing the perceived threat of participation in physical activity with a DFU may be a first and important step in people with a DFU meeting recommended levels of daily physical activity, engaging in exercise and subsequently improving their health status.

The short exercise intervention trialled in the current study did not result in a change in overall physical activity levels from the IPAQ survey, nor perceived barriers to exercise as assessed by the EBBS. Conversely, we did note a reduction in the perceived benefits of exercise. As there is a no documented minimally clinically important difference for this scale that we were able to find, we cannot determine if this change is clinically relevant. Satisfaction with the exercise programme was acceptable as recorded by the satisfaction survey. There is a possibility of participant bias in these results. Best efforts to control for this included ensuring participants knew data was confidential and providing the survey electronically to optimise anonymity. small sample size bias is also likely to have impacted these results.

Behaviour modification interventions have been trialled in studies of people with diabetes but without DFU with some success. These types of interventions may have applicability in the DFU population. A literature review and practice guideline outlined that the inclusion of behaviour change interventions, with a focus on self-efficacy and motivation to exercise, could be effective when added to exercise to increase physical activity levels in people with diabetes [34]. A study by Olson et al. (2015) [35] incorporated group workshops for goal setting and behaviour modification strategies in combination with walking programmes. Although long term behaviour change was not demonstrated in this study, the combination of psychological and physical interventions demonstrated short term success for increasing participant’s physical activity levels. Whilst promising in concept, the applicability of such a programme in people with DFU would need further investigation.

Another interesting finding in this study was the disproportionately high numbers of participants that lived regionally or rurally and were unable to attend in-person follow up sessions. This is a unique situation for Western Australia where the population is spread over an area of more than two-million square kilometres. It presents a challenge when delivering healthcare, particularly when using supervised or group exercise as a potential treatment modality. Provision of a home exercise programme rather than utilising an in-person supervised exercise environment is one way of combatting this, however innovation and improvements in healthcare delivery, including the ongoing use of telehealth modalities to monitor performance, are necessary to improve healthcare outcomes.

### Limitations

The availability of funding and personnel limited the undertaking of recruitment and intervention to one day per week for the duration of this study. This limited our ability to recruit participants who were unwell or fasting for surgical intervention on that scheduled day of recruitment. Therefore, if a patient was unwell on that day, they may not have had another opportunity to participate prior to discharge from hospital. Similarly, if a patient was for discharge on that day, they were more likely to decline participation. As such, our recruitment rate was short of our pre-planned acceptability level.

Another challenge for ongoing follow-up of participants was the government issued travel and hospital visitation restrictions associated with the COVID-19 pandemic. Our method for mitigation of this challenge was utilising virtual or telehealth modes of communication which enabled us to achieve high levels of survey completion in the follow up period. We were unable to use telehealth to provide home exercise supervision or monitoring of compliance for this project. This was reliant on demonstration or exercises on initial review and patient report of compliance throughout.

### Future research

This pilot study for feasibility and safety of exercise will be used to inform future research design. Future randomised trials in this population should have larger sample size with longer duration intervention and include outcomes of both the foot and musculoskeletal system.

Telehealth models of care should be considered for providing supervision of home exercise programmes to increase compliance with an exercise intervention. Patients who decline participation in an exercise research trial should be invited to provide outcome data only for comparison with trial participants, as well as inclusion in any qualitative study to explore barriers to exercise and participation.

## Conclusion

Exercise appears safe to be undertaken by patients with diabetes related foot ulcers during a hospital admission. Recruitment in this acute setting proved a challenge in this study due to clinical demands in the acute setting, but adherence, retention and satisfaction with participation in exercise met our pre-determined acceptable limits.

## Supplementary Information

**Additional file 1.** Home Exercise Programme. This PDF file is an example of the home exercise programme provided to participants. © South Metropolitan Health Service Western Australia 2021. Copyright to this material is vested in the State of Western Australia unless otherwise indicated. Apart from any fair dealing for the purposes of private study, research, criticism or review, as permitted under the provisions of the *Copyright Act 1968*, no part may be reproduced or re-used for any purposes whatsoever without written permission of the State of Western Australia.

## Data Availability

The deidentified datasets used and/or analysed during the current study are available from the corresponding author on reasonable request.

## Abbreviations

DFU: Diabetes related foot ulcer
IWGDF: International Working Group on the Diabetic Foot
REDCap: Research Electronic Data Capture
BGL: Blood glucose level
CR-10: Borg’s Rating of Perceived Exertion Category-Ratio scale
IPAQ: International Physical Activity Questionnaire
EBBS: Exercise Benefits Barriers Scale
RPE: Rating of perceived exertion
sRPE: Session rating of perceived exertion
BMD: Bone mineral density
