# Supplementary material for: Exercise in adults admitted to hospital with diabetes-related foot ulcers: a pilot study of feasibility and safety
Source: J Foot Ankle Res. 2023 Mar 28;16:18. doi: 10.1186/s13047-023-00616-0 (PMC10043540; doi:10.1186/s13047-023-00616-0)
Supplement: Supplementary file 1 — Additional file 1. Home Exercise Programme. This PDF file is an example of the home exercise programme provided to participants. © South Metropolitan Health Service Western Australia 2021. Copyright to this material is vested in the State of Western Australia unless otherwise indicated. Apart from any fair dealing for the purposes of private study, research, criticism or review, as permitted under the provisions of the Copyright Act 1968, no part may be reproduced or re-used for any purposes whatsoever without written permission of the State of Western Australia. [file 13047_2023_616_MOESM1_ESM.pdf]

Exercising with your diabetes related foot ulcer

Exercise is very important to your health. National guidelines recommend all adults complete 2–5 hours of exercise per week (30 minutes per day), including two strength exercise sessions.

It can be difficult to meet these guidelines, however, if you have been advised to stay off your foot to help your diabetes related ulcer heal.

The exercises featured in this guide can be completed at home with minimal equipment. This resource is a general guide only, so you may need to change or leave out some of the exercises if they are too hard or too easy.

If you would like personal advice about exercises you can either:

- see your local physiotherapy practice
- ask your GP to set up a General Practitioner Management Plan so you can access a physiotherapist.

Patient name:

Notes:

Date:

Before you exercise

Stay safe

It is important to monitor your blood glucose levels before and after exercise.

Do not exercise if:

- your blood glucose level is less than 5mmol/L
- you are a Type 1 diabetic, and your blood glucose is above 15mmol/L, and you have ketones.

Remember to:

- monitor your feet each day for changes in your ulcer or new ulcers – if you can't monitor your own feet, ask someone else to look for you
- change your socks after exercise to avoid dressings getting wet.

You can always chat with your doctor, podiatrist, or physiotherapist if you have any concerns.

Equipment needs

Make sure you have the following ready before your exercise:

- a chair
- weights (hand weights, filled up water bottles, tins of food from the cupboard)
- theraband (a stretchy exercise band you can buy at pharmacies or department stores)
- a drink of water to stay hydrated
- prescribed footwear or shoes.

When and how often to exercise

Aim to complete these exercises 2–3 times per week.

How much exercise you do also depends on how hard you find each exercise. Examples of how many of each exercise you should try to complete are written under each exercise. You should work until your muscles feel tired as this is the only way you will become stronger.

To make exercises harder you can:

- add weights or theraband to the exercise
- complete the exercise slower
- complete more of each exercise or do more cycles of the program.

After exercise

It is normal to feel some muscle soreness after completing the exercise program, especially the first few times or when you increase weights or amounts of exercises. Whilst it can be unpleasant for a day or two, it is usually a good sign that you have worked your muscles hard.

It is also normal for your breathing to get a bit harder and your heart rate to increase a little.

If you experience any chest pain, severe shortness of breath or dizziness during exercise, stop immediately and seek urgent medical attention or dial 000 for an ambulance if these symptoms persist.

Your exercise program

This guide is divided into sections:

- arm and leg exercises for everyone
- leg exercises for people who have been advised to:
  - weight bear through their heel (for ulcers at the front of the foot)
  - weight bear through their toe (for ulcers at the back of the foot)
  - not to weight bear on one foot at all (for particularly bad ulcers).

Your podiatrist or doctor can help you complete the section below to determine which exercises are right for you.

My weight bearing status is:

- ☐ heel weight bearing (for ulcers at the front of your foot)
- ☐ toe weight bearing (for ulcers at the back of your foot)
- ☐ non-weight bearing (for particularly bad ulcers)

My specialised footwear is:

- ☐ Darco shoe
- ☐ Camboot
- ☐ other

Tip: to remember which exercises section to follow, mark the relevant exercises with a highlighter or cross out sections that don't apply to you.

Warmup exercises

A warmup is useful to get blood flowing around your body and get your muscles warm and ready for exercising.

Choose one of the options below depending on if you have access to a stationary bicycle and what your weight bearing status is. Cross out the options that aren't appropriate for you.

**Air punching**<br><br>Punch forwards into the air in front of you, adding weights (or water bottles/ tins from the cupboard) in your hands to make this harder. Aim for 2–5 minutes.	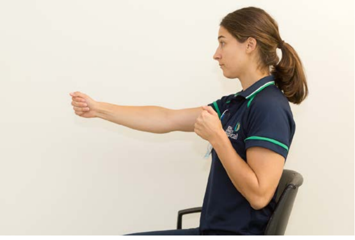	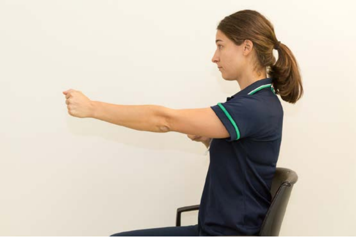
**Cycling with heel weight bearing**<br><br>Make sure you only put weight through the heel of your affected foot. Aim for 5–15 minutes.	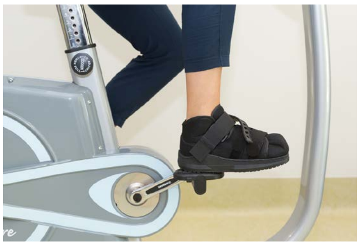	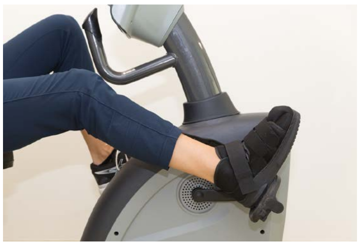
**Cycling with toe weight bearing**<br><br>Make sure you only put weight through the toe of your affected foot Aim for 5–15 minutes.	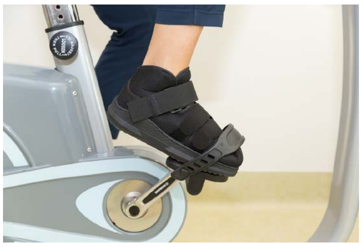	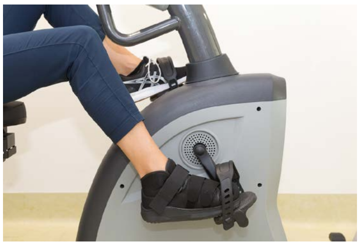
**Cycling with one leg**<br><br>Make sure you only cycle using the unaffected leg and are securely balanced. Aim for 5–15 minutes.	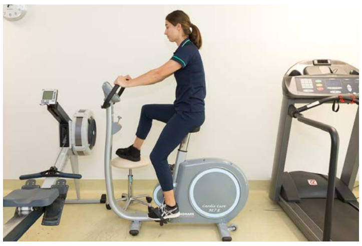	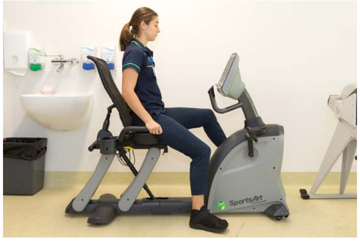

Exercises for everyone

The arm exercises below can be done regardless of the weight bearing status of your foot. If any of the exercises cause shoulder soreness, try using smaller weights or smaller movements, or cross the exercise off your program.

Seated bicep curls

Sit with your arms relaxed down beside you, holding weights.

If you are sitting in a chair with arms, you may need to wriggle a bit closer to the edge.

Start with palms facing out, then lift the weights by bending your elbows until your hands are near your shoulders. Slowly lower.

Aim for 10–20 times on each arm, 2–3 sets.

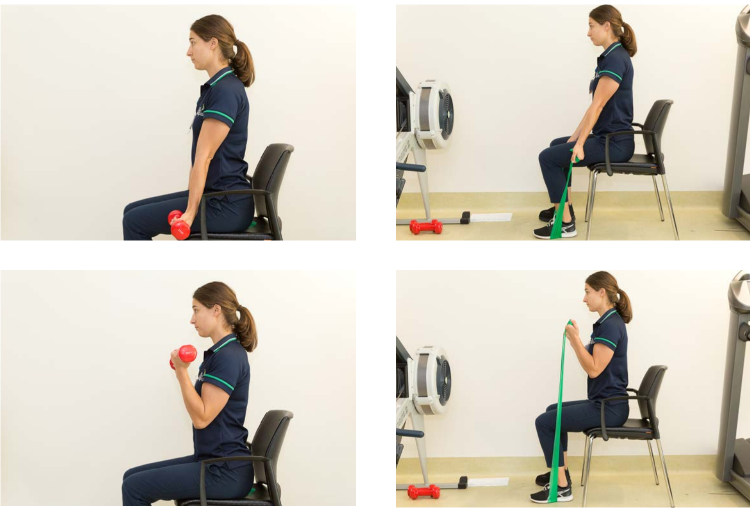

Seated bicep curls with weights, and with theraband.

Theraband row

Sit in front of a sturdy object (for example the leg of a heavy table or a pole). Loop the theraband around the sturdy object.

Pull your hands backwards by squeezing your shoulder blades together. Return to the starting position in a controlled manner.

Aim for 10–20 times on each arm, 2–3 sets.

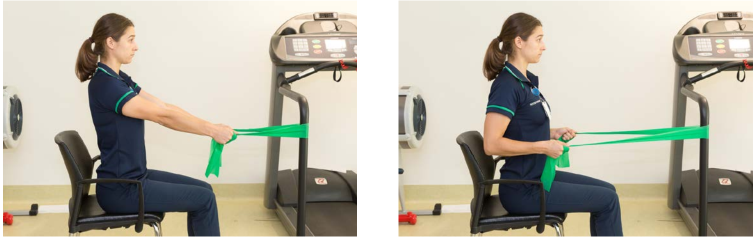

Theraband row

Shoulder press

Sit in a chair, with weights in your hands.

Start with your elbows bent and your hands near your shoulders.

Press your hands up towards the ceiling, and then slowly lower back to the starting position.

Aim for 10–20 times on each arm, 2–3 sets.

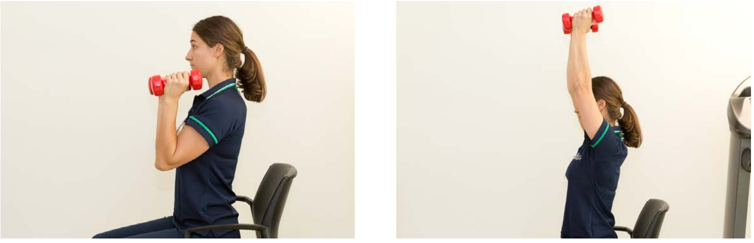

Shoulder press

Lateral arm raises

Sit with your arms relaxed down beside you, holding weights.

If you are sitting in a chair with arms, you may need to wriggle a bit closer to the edge.

Lift your arms outwards, as high as is comfortable. Slowly lower back to the starting position.

Aim for 10–20 times on each arm, 2–3 sets.

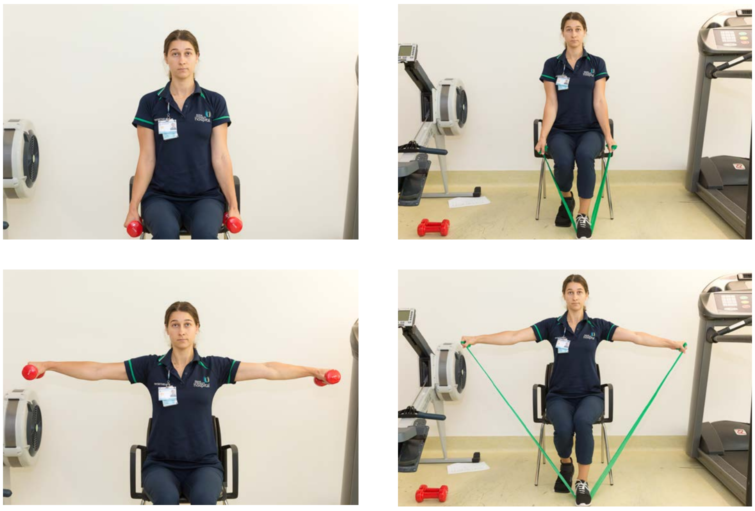

Lateral arm raises with weights, and with theraband

The following leg exercises can be done regardless of the weight bearing status of your foot.

Knee extensions

Sit on a chair with theraband looped around your ankle and the leg of the chair as shown, or with ankle weights strapped to your ankles if you have these. Ensure the band or weight is not on or near your ulcer.

Slowly straighten your leg against the resistance, hold and then slowly return to the starting position.

Aim for up to 25 times each leg, 2–3 sets.

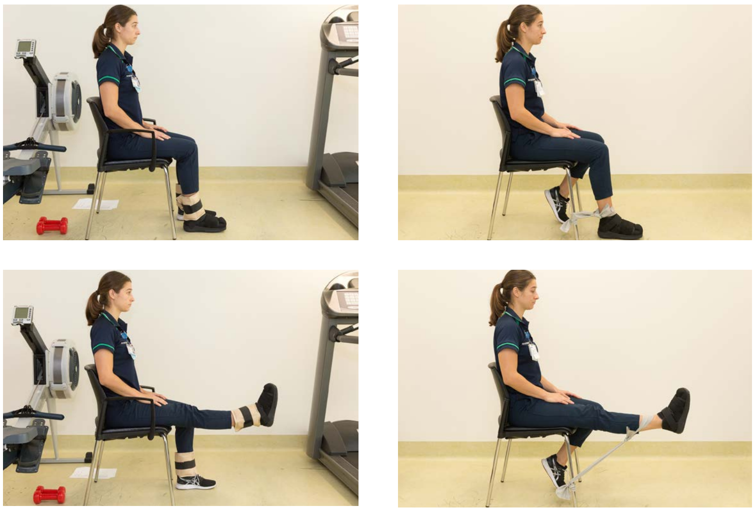

Knee extensions with ankle weights, and with theraband

Side lying leg raises

Lie on one side. Your bottom leg can be bent for balance, and your top leg should be straight.

Lift your top leg up towards the roof, trying to keep your knee pointing forwards. Hold, and then slowly lower. You can add an ankle weight or tie the theraband around your legs to make this harder.

Aim for 10–20 times on each leg, 2–3 sets.

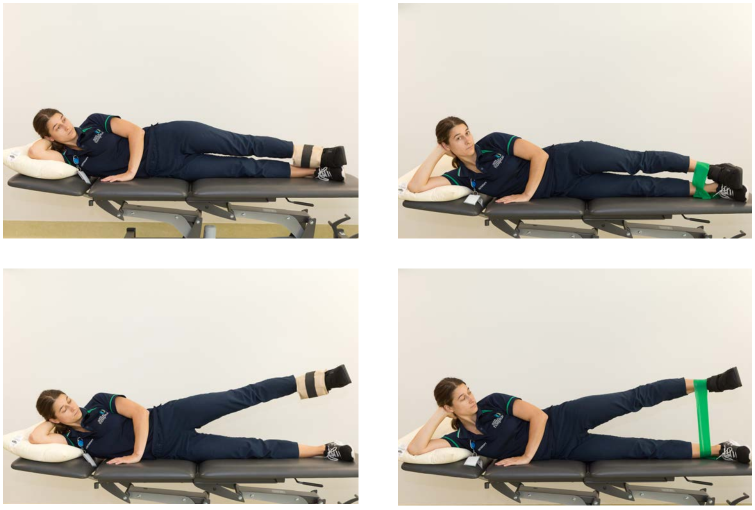

Side lying leg raises with ankle weights, or theraband

Hip extensions on your stomach

Lie on your stomach with the resistance band around both thighs or weights on each ankle. Without twisting your hips or back, squeeze your buttock and lift one leg as high as you can toward the ceiling. Slowly lower. Repeat exercise with your other leg.

Aim for 10-20 times on each leg, 2–3 sets.

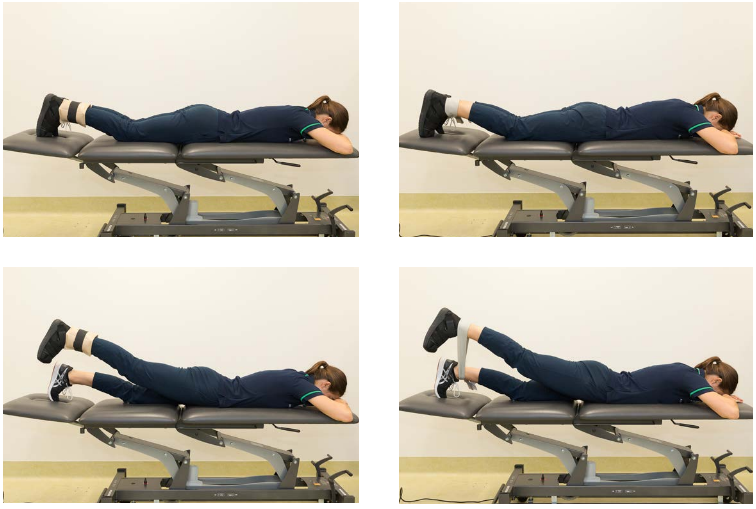

Hip extensions with ankle weights, or theraband

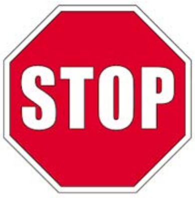

Depending on the location of your foot ulcer, **only one of the following three sets of leg exercises will be appropriate for you.**

If you are unsure, ask your physiotherapist, doctor, or podiatrist.

Tip: to remember which exercise section to follow, mark the relevant exercises with a highlighter or cross out exercises that don't apply to you.

Heel weight bearing exercises

Make sure there is no weight going through the front of your foot to protect your ulcer. If you cannot do an exercise without putting weight through the ulcer, cross it off your program.

Bridging with heel weight bearing

Lie on your back in your bed or on the floor if you find it easy to get up and down from the floor.

Bring your heels in close to your buttocks and lift your toes up so just your heels are in contact with the bed or floor.

Squeeze your buttocks to lift upwards at the hips, hold and then slowly lower.

Aim to do 10–15 times, 2–3 sets.

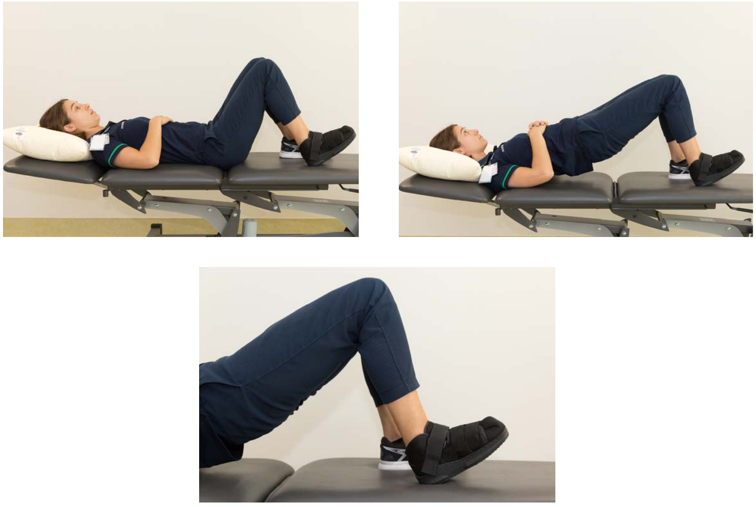

Bridging with heel weight bearing

Sit to stand with heel weight bearing

Sit on a chair, with your bottom close to the front. The foot with the ulcer should be out in front a bit more, with your toes lifted.

Push up to standing, using your arms as much as needed. To make it harder, try not to use your arms as much, or not at all. Slowly lower down.

Aim to do 5–15 times, 2–3 sets.

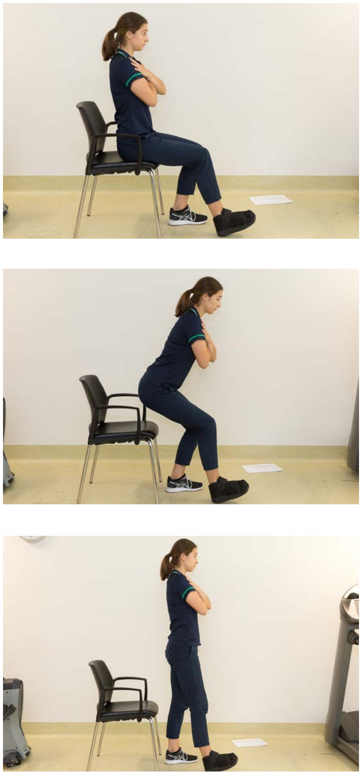

Sit to stand with heel weight bearing without arms

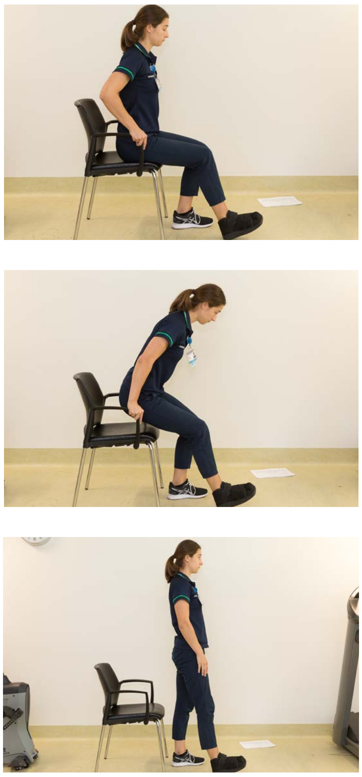

Sit to stand with heel weight bearing with arms

Toe weight bearing exercises

Make sure that there is no weight going through the heel of your foot, to protect your ulcer. If you cannot do an exercise without putting weight through the ulcer, cross it off your program.

Heel raises

Stand in front of a bench or table, and take support with your arms for your balance.

Push up through your toes so that your heels lift off the ground, hold and then slowly lower back down.

Aim to do up to 25 times, 2–3 sets.

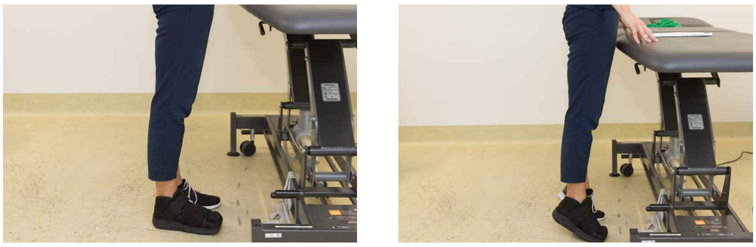

Heel raises

Exercises if you can't weight bear on one foot

Make sure your foot is off the ground, to protect your ulcer. If you cannot do an exercise without putting weight through the ulcer, cross it off your program.

Single leg bridging

Lie flat on your bed. Bring the foot you are allowed to weight bear on towards your buttocks. Your non weight bearing leg can either be lifted into the air or crossed over your other leg as shown (this is slightly easier).

Squeeze your buttocks to lift upwards at the hips, hold and then slowly lower.

Aim to do 5–15 times, 2–3 sets.

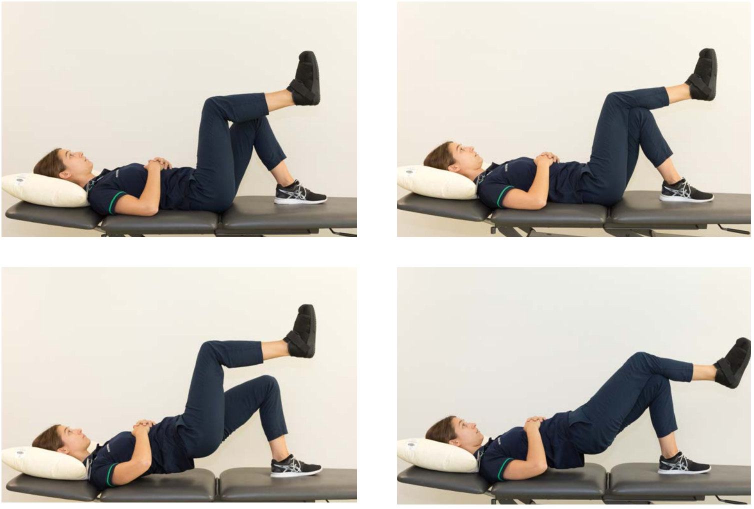

Single leg bridging, or single leg bridging with leg cross

Learn more about diabetes

Visit Diabetes Australia for more information on exercise and how to manage your diabetes.
www.diabetesaustralia.com.au
Email: **info@ndss.com.au**
Phone: **1800 637 700**

More information

If you have questions about this information sheet, please phone the **Endocrinology Physiotherapist** on _______________.

This document can be made available in alternative formats on request.

Compiled: Fiona Stanley Fremantle Hospital Group, Physiotherapist
Review: August 2021 Next review: August 2024
Publication number - 2021-SMHS-765
© State of Western Australia, South Metropolitan Health Service 2021.

CONSUMER REVIEWED
